# Supplementary material for: Gene‒Prostate-Specific-Antigen-Guided Personalized Screening for Prostate Cancer
Source: Genes (Basel). 2019 Aug 24;10(9):641. doi: 10.3390/genes10090641 (PMC6770934; doi:10.3390/genes10090641)

Supplementary text: Risk scores for initiators and promoters for prostate cancer

$$
\begin{aligned}
\text{Risk score 1} \; = {} & 0.9394 \times (\text{Age } 59 - 62) + 1.3757 \times (\text{Age } 63 - 66) \\
& + 1.8034 \times (\text{Age} > 66) + 0.1398 \times (\text{PSA } 4.01 - 5.0) \\
& + 0.7655 \times (\text{PSA } 5.01 - 6.0) + 0.7324 \times (\text{PSA } 6.01 - 8.0) \\
& + 0.9002 \times (\text{PSA } 8.01 - 10.0) + 1.2669 \times \; (\text{PSA } 10 +) \\
& + 0.4978 \times \big(\text{rs4242382(AA)}\big) + 0.10838 \times \big(\text{rs4242382(GA)}\big) \\
& + 0.3221 \times (rs4430796) + 0.2468 \times (rs1859962) \\
& + 0.4253 \times (rs16901979) + 0.3184 \times (rs6983267) \\
& + 0.1988 \times (rs1447295) + 0.0769 \times (\text{rs2660753}) \\
& + 0.1310 \times (\text{rs9364554}) + 0.1132 \times (\text{rs6465657}) \\
& + 0.2231 \times (\text{rs10993994}) + -0.1625 * (\text{rs7931342}) \\
& + -0.1165 \times (\text{rs2735839}) + 0.2546 \times (\text{rs5945619}) \\
& + 0.1397 \times (\text{rs721048}) + -0.3011 \times \big(\text{rs10486567 (GG)}\big) \\
& + -0.3424 \times \big(\text{rs10486567 (GA)}\big) + 0.6471 \times (\text{family history})
\end{aligned}
$$

$$
\begin{aligned}
\text{Risk score 2} \; = {} & 2.0643 \times (\text{rs200331695}) + 1.1631 \times \big(\text{IGF} - \text{I}_{Q2}\big) \\
& + 1.2528 \times \big(\text{IGF} - \text{I}_{Q3}\big) + 1.6292 \times \big(\text{IGF} - \text{I}_{Q4}\big) \\
& + 1.5151 \times (\text{GSTP1 hypermethylation})
\end{aligned}
$$

Supplementary Figure 1. The cumulative risks of high-grade PrCa and PrCa death for patients with different PSA levels and genetic risk

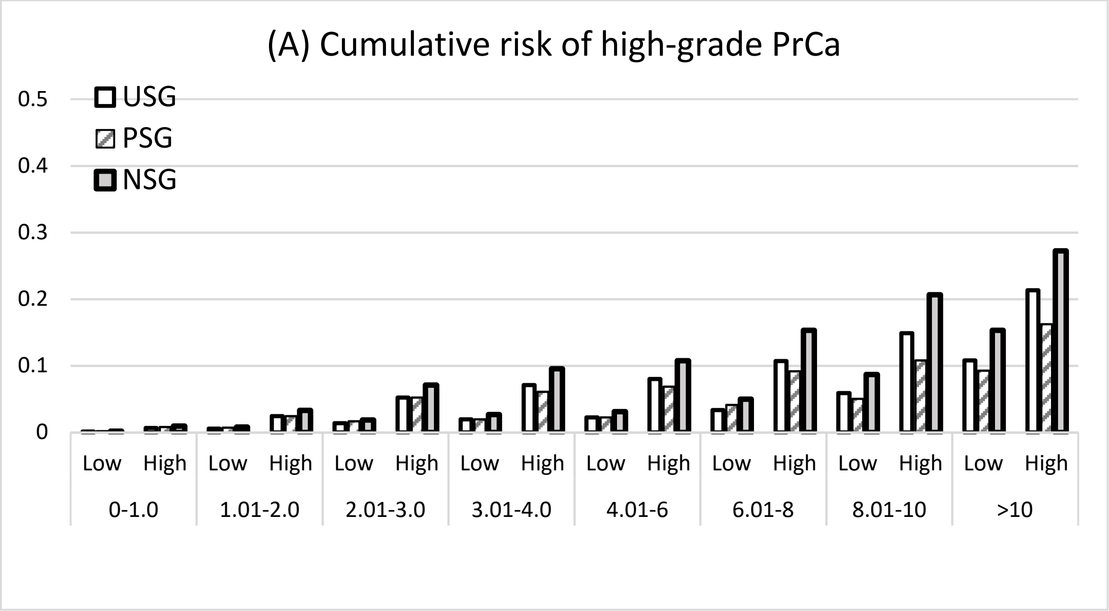

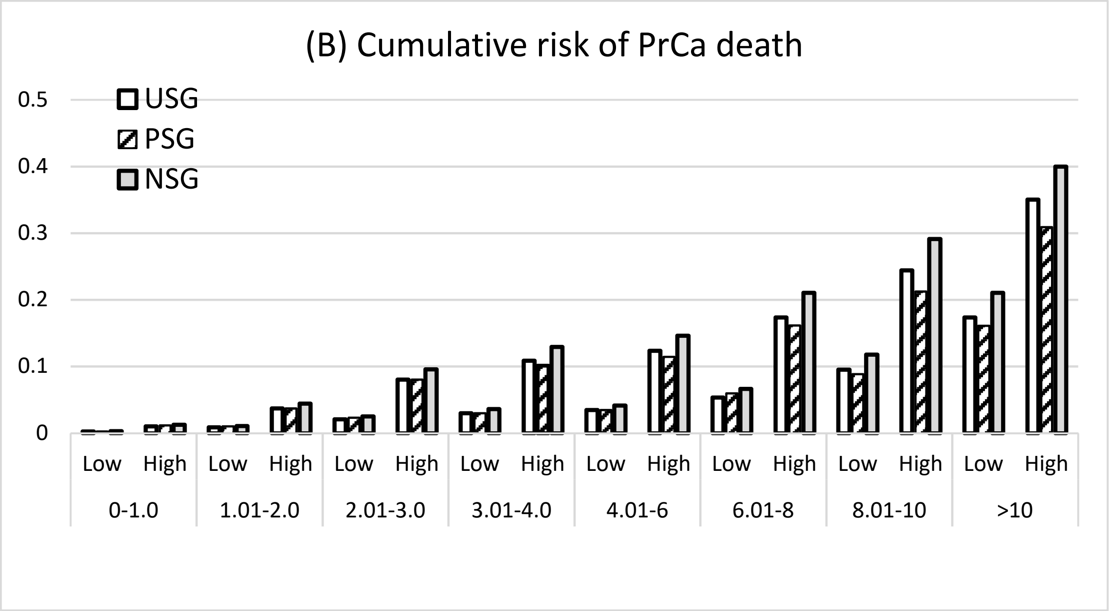

Supplement: Supplementary file 1 [file genes-10-00641-s001.pdf]
